# Assessing the Desmoid-Type Fibromatosis Patients' Voice: Comparison of Health-Related Quality of Life Experiences from Patients of Two Countries

**DOI:** 10.1155/2020/2141939

**Published:** 2020-07-26

**Authors:** Milea J. M. Timbergen, Winette T. A. van der Graaf, Dirk J. Grünhagen, Eugenie Younger, Stefan Sleijfer, Alison Dunlop, Lucy Dean, Cornelis Verhoef, Lonneke V. van de Poll-Franse, Olga Husson

**Affiliations:** ^1^Department of Surgical Oncology, Erasmus MC Cancer Institute, Rotterdam, Netherlands; ^2^Department of Medical Oncology, Erasmus MC Cancer Institute, Rotterdam, Netherlands; ^3^Department of Medical Oncology, The Netherlands Cancer Institute, Amsterdam, Netherlands; ^4^Department of Medical Oncology, Radboud University Medical Centre, Nijmegen, Netherlands; ^5^Sarcoma Unit, Royal Marsden NHS Foundation Trust, London, UK; ^6^Division of Clinical Studies, Institute of Cancer Research, Royal Marsden NHS Foundation Trust, London, UK; ^7^Division of Psychosocial Research and Epidemiology, The Netherlands Cancer Institute, Amsterdam, Netherlands; ^8^Department of Medical and Clinical Psychology, Tilburg University, Netherlands; ^9^Department of Research, Netherlands Comprehensive Cancer Organisation (IKNL), Eindhoven, Netherlands

## Abstract

**Purpose:**

Desmoid-type fibromatosis (DTF) is a rare, nonmetastasising soft tissue tumour. Symptoms, unpredictable growth, lack of definitive treatments, and the chronic character of the disease can significantly impact health-related quality of life (HRQoL). We aimed at identifying the most important HRQoL issues according to DTF patients in two countries, in order to devise a specific HRQoL questionnaire for this patient group.

**Methods:**

DTF patients and healthcare providers (HCPs) from the Netherlands and the United Kingdom individually ranked 124 issues regarding diagnosis, treatment, follow-up, recurrence, living with DTF, healthcare, and supportive care experiences, according to their relevance. Descriptive statistics were used to calculate priority scores.

**Results:**

The most highly ranked issues by patients (*n* = 29) were issues concerning “tumour growth,” “feeling that there is something in the body that does not belong there,” and “fear of tumour growth into adjacent tissues or organs” with mean (*M*) scores of 3.0, 2.9, and 2.8, respectively (Likert scale 1–4). British patients scored higher on most issues compared to Dutch patients (*M* 2.2 vs. *M* 1.5). HCPs (*n* = 31) gave higher scores on most issues compared to patients (*M* 2.3 vs. *M* 1.8).

**Conclusion:**

This study identified the most relevant issues for DTF patients, which should be included in a DTF-specific HRQoL questionnaire. Additionally, we identified differences in priority scores between British and Dutch participating patients. Field testing in a large, international cohort is needed to confirm these findings and to devise a comprehensive and specific HRQoL questionnaire for DTF patients.

## 1. Introduction

Sporadic desmoid-type fibromatosis (DTF) is a rare, borderline tumour of the soft tissues [1–3]. Most patients are females, aged between 20 and 40 years at primary diagnosis [3]. Sporadic DTF arises in musculoaponeurotic structures with the most common sites being the abdominal wall and the extremities [4]. Symptoms vary, depending on tumour site, size, and infiltration of adjacent structures, resulting in pain and/or functional impairment. DTF does not metastasize, rarely has fatal outcomes, often displays long periods of spontaneous stabilisation, and can undergo spontaneous regression [5]. Surgical resection, radiotherapy, and noncytotoxic and cytotoxic systemic therapies may be considered in patients with symptomatic disease, but unfortunately, these “traditional” treatment options do not guarantee tumour reduction and/or clinical response [6]. Local recurrence after surgery remains high [7, 8], leading to a reduction in surgical treatments for DTF over recent decades [3, 4]. Additionally, “active” forms of treatment can be debilitating, causing greater morbidity than the tumour itself. For these reasons, active surveillance is now recommended as a first-line management for most patients with DTF [6, 9]. Therefore, DTF has obtained a “chronic” status and its impact on patients should be evaluated accordingly.

Health-related quality of life (HRQoL) provides information beyond traditional measures of efficacy in oncology such as overall survival and is increasingly used as an endpoint in clinical trials [10, 11]. We previously performed a systematic literature review to evaluate which HRQoL measures were used in research to assess HRQoL in DTF [12]. Generic HRQoL measures (e.g., the cancer-specific core questionnaire from European Organisation for Research and Treatment of Cancer; the EORTC Quality of Life Core Questionnaire (EORTC QLQ-C30)) may not consider disease-specific issues in DTF patients. Site-specific tools (e.g., Toronto Extremity Salvage Score) may not be relevant to certain groups (e.g., those with abdominal wall or head and neck tumours).

At present, there is no validated DTF-specific HRQoL tool, and this was illustrated by a systematic literature review published by our group [12, 13]. In order to gain greater insight into the issues that patients with DTF experience in their daily lives, and to evaluate their experiences of healthcare including the supportive care system, we previously organised focus groups and semistructured interviews, in the United Kingdom (UK) and in the Netherlands (NL) [12, 13]. These studies identified issues covering various domains including the diagnostic pathway, the treatment pathway, daily limitations (e.g., physical and psychological symptoms), and experiences with the current healthcare system.

The main goal of this study was to determine the relative importance of each issue and receiving feedback on the appropriateness of content and breadth of coverage. In the present study, we used the previously identified issues to (1) identify the most relevant issues to patients with DTF in two healthcare settings (UK and NL) and to (2) identify differences in scores between both countries.

## 2. Materials and Methods

### 2.1. Identification of Issues

The EORTC Quality of Life Group methodology for developing a questionnaire was used for the selection of relevant issues based on previous focus groups and patients interviews [14]. Issues that had previously been identified to be of concern to DTF patients were listed per country (UK and NL). A total of 188 issues were identified in the UK group and 110 issues were identified in the Dutch group. Next, issues were grouped into categories and duplicate issues covering the same topics were removed. A total of 124 issues were converted into a provisional list of issues. All issues were reviewed by two authors (MT and OH). All issues were translated by native English and Dutch speakers.

### 2.2. Patient Selection

Patients with DTF were approached for participation by their treating physician. Inclusion criteria were histologically proven DTF, age ≥18 years, Dutch or English language skills, and a “recent” visit (<2 years) to the hospital. Exclusion criteria were participation in one of the previous focus groups or patient interviews and patients with a diagnosis of cancer or familial adenomatous polyposis (FAP). Patients received an information letter which explained study objectives. Baseline characteristics and details about the individual disease trajectory of participants were obtained. Patients were only invited to participate once and did not have to provide a reason if they declined. No reminders were sent. All data from patients was collected and processed anonymously.

### 2.3. Selection of Healthcare Providers

To examine whether HCPs with expertise and experience in sarcomas and DTF have the same perspectives as patients with DTF about key HRQoL issues, an e-survey of the same 124 issues was created using LimeSurvey Servicebedrijf© software. The issue list was available in two languages (Dutch and English), and issues were presented in a random order. In the Netherlands, HCPs from the multidisciplinary team (e.g., surgeons, oncologists, radiologists, radiotherapists, sarcoma clinical nurse specialists, and physiotherapists) were identified using the website Orphanet, which provides information on centres of expertise dedicated to the medical management for rare diseases (https://www.orpha.net/consor/cgi-bin/Clinics_Search.php?lng=EN). In the UK, HCPs of the aforementioned disciplines were identified using the sarcoma network group of the Royal Marsden Hospital, London, UK. Every HCP received an invitation email with a token and link to the e-survey. A reminder was sent after one week if the HCP had not responded.

### 2.4. Sociodemographic and Clinical Characteristics

Age at the time of diagnosis was either stated by the patient or calculated using the date of birth and date of the first pathology report. Age at the time of questionnaire completion was either stated by the patient or calculated using the date of informed consent and the date of birth. Education levels were categorized into “high” (PhD, university, and higher education postgraduate/undergraduate degree), “intermediate” (professional qualification, vocational work, work-related qualification, general secondary education, and further/intermediate education), and “low” (primary education (with a higher, but not completed education) and secondary education). Continuous variables were presented as a mean with a standard deviation (SD) or as a median with an interquartile range (IQR). Categorical variables were presented as number (*n*) using frequencies and percentages.

### 2.5. Presentation of Issues to Patients and Healthcare Providers

A total of 124 issues were presented to patients and healthcare providers (HCPs) in a random order (Supplemental Table 1). Patients and HCPs scored 124 issues by relevance on a Likert scale from 1 to 4 ((1) not at all, (2) a little, (3) quite a bit, and (4) very much) and ranked the top ten most important issues. The frequency that each issue appeared in the top ten most important issues was converted into the mean priority score (*M*-score) per issue. The frequency of the top ten priority score of each issue was calculated and ranked in overall priority score. Where questions were left blank by the participant, they were coded as a “missing value” and not incorporated in the total score. Space for general remarks was available at the end of the questionnaire.

### 2.6. EORTC QLQ-C30 Questionnaire

In addition to the issue list, patients were asked to fill out the 30-item EORTC QLQ-C30 questionnaire (version 3) to assess HRQoL [15]. Norm data were obtained from the EORTC, which recently collected data from the general population in Europe and North America [16]. Only data from the general population in the Netherlands and the UK were used for the current study. The EORTC QLQ-C30 questionnaire contains five functional scales (physical, role, cognitive, emotional, and social functioning), a global health status scale, three symptom scales (fatigue, nausea and vomiting, and pain), and six single items (appetite loss, diarrhoea, dyspnoea, constipation, insomnia, and financial difficulties). The questionnaire has a 1-week time frame and uses a four-point response format (“not at all,” “a little,” “quite a bit,” and “very much”), with the exception of the global health status scale, which has a seven-point response format. The scores were calculated using linear transformation to a score between 0 and 100. For the functional scales and the global health status, a high score represents a high (healthy) level of functioning. A high score for the symptom scales represents a high level of symptoms (greater symptom burden) [17]. The EORTC QLQ-C30 summary score was calculated using the mean scores of the function scales and the reversed mean scores of the symptom scales and single items (financial impact and global health status excluded) and is summarized as the mean of the combined 13 QLQ-C30 scale scores. A higher summary score represented a better outcome [18, 19]. The summary score was only calculated when all of the required 13 scale and item scores were available. Data analysis and handling of missing items were done according to the scoring manual of the EORTC [17].

### 2.7. Statistical Analysis

Patients were matched, using a 1 : 10 nearest-neighbour match method, with the general population based on nationality, age, and sex using RStudio (RStudio, version 1.0.153, Boston, MA, package MatchIt). Patients with missing values (lacking information regarding age or sex) were excluded from the analysis. Differences in priority scores (Dutch versus British participating patients and HCPs versus participating patients) and differences in scores of the EORTC QLQ-C30 scales between groups (Dutch versus British participating patients and Dutch and British participating patients versus the Dutch and British general population) were tested for their significance using the Mann–Whitney *U* test. SPSS Statistics (version 24) was used for the Mann–Whitney *U* tests (IBM, Armonk, New York, USA). Two-sided *p* < 0.05 was considered statistically significant.

## 3. Results

### 3.1. Patient Cohort

**Figure 1:**
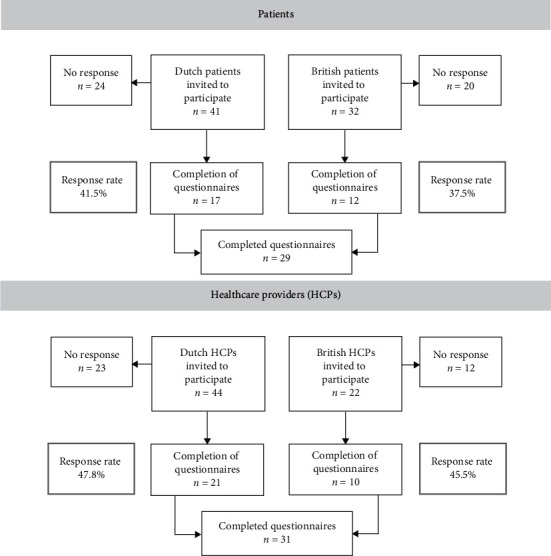
Flow diagram of participating patients and healthcare providers' responses to this survey.

Forty-one patients from the Erasmus MC, Rotterdam, the Netherlands, and 32 patients from the Royal Marsden Hospital, London, UK, were approached during July and August 2018. Out of 73 patients, 29 patients (total response rate of 39.7%) gave written informed consent (Figure 1). The cohort comprised of 10 males and 19 females with DTF most commonly localized in the extremities, flank, and chest wall (*n* = 15, 52%). Nine participants had received active treatment at the time of the questionnaire. The median, self-reported age at diagnosis was 38 years (IQR 30–48) (Table 1). Sociodemographic characteristics are summarized in Supplemental Table 1. All participants completed the issue list, and sixteen participants ranked their top 10 most relevant issues.

### 3.2. Ranking of Priority of the Issues

Ranking of HRQoL issues revealed that 13 out of 124 issues (10.5%) were chosen to be the most relevant (prevalence ratio of >30%) (Table 2). Patients considered the following issues as relevant and missing on the current issue list: “problems with healthcare insurances,” “coverage of costs related to the disease such as traveling costs,” “lack of adequate online information,” “lack of knowledge about treatment options outside the region or country,” “lack of information about pain management and referral to pain professionals,” and “lack of advice regarding dietary restrictions or playing sports.” A list of the missing items, general remarks, and quotes is provided in Supplemental Tables 3 and 4.

### 3.3. British versus Dutch Patients

Overall, British patients gave higher scores for each issue compared to Dutch patients (*M*-score 2.2 (UK) vs. *M*-score 1.5 (NL)) (Supplemental Table 2). Differences in score of more than 1 point between Dutch and British patients are displayed in Supplemental Figure 1. Additionally, priority scores of Dutch and British HCPs and scores of participating patients and HCPs from the Netherlands and the UK were compared (Supplemental Table 2). The total cohort of patients was too small to identify any differences between subgroups (e.g., initial treatment type, tumour location, and age at diagnosis).

### 3.4. Healthcare Providers

In the Netherlands, HCPs were invited to six sarcoma centres. All HCPs from the UK were employees at the Royal Marsden Hospital, London. Twenty-one Dutch and ten British HCPs responded. Professional backgrounds included surgical oncologist (*n* = 12), medical oncologist (*n* = 6), radiation oncologist (*n* = 5), specialized sarcoma nurse (*n* = 5), and other professions including a radiologist, physiotherapist, and pain specialist (all *n* = 1). Seventeen professionals had more than 10 years of experience, three had 6–10 years of experience, and eleven had 5 or less years of experience working with desmoid patients. Frequency of contact with DTF patients varied between once a week (*n* = 9, 29%) to rarely (less than once every 3 months) (*n* = 1, 3%).

Issues with the highest scores according to HCPs included “worries about tumour growth” (*M*-score 3.4), “stress about the diagnosis” (*M*-score 3.2), “the experience of uncertainty during the course of the disease” (*M*-score 3.2), “pain” (*M*-score 3.2), “concerns about the future” (*M*-score 3.0), “stress around check-ups during the follow-up” (*M*-score 3.0), “fear of recurrence after treatment” (*M*-score 3.0), “fear of tumour growth/tumour growth into adjacent tissues or organs” (*M*-score 2.9), and “the feeling that patients do not have a clear prognosis” (*M*-score 2.9). Overall, HCPs from the UK gave higher scores, compared to Dutch HCPs with *M*-scores of 2.8 and 2.0, respectively (Supplemental Table 2).

### 3.5. Participating Patients versus Healthcare Providers

There was considerable overlap between the highest ranked issues according to patients and HCPs, particularly regarding the unpredictable growth pattern of DTF tumours (Supplemental Table 1). HCPs scored significantly higher (*p* < 0.05) on 77 out of a total of 77 of 124 issues. HCPs also gave a higher mean overall score on the issues list (total *M*-score 2.3) compared to patients (total *M*-score 1.8) (Supplemental Table 2).

### 3.6. EORTC QLQ-C30: Dutch vs. British Participating Patients

Overall, the mean summary score for the EORTC QLQ-C30 for all DTF patients together was 78.1, with a mean global health score of 68.7 (Table 3). Statistically significant differences between scores of British and Dutch patients were found for “global health,” “insomnia,” for the symptom scales “pain” and “fatigue,” and for the following functioning scales “cognitive functioning,” “emotional functioning,” “social functioning,” and “role functioning” (Table 3).

### 3.7. EORTC QLQ-C30: Participating Patients versus the Matched General Population

After 1 : 10 nearest-neighbour matching based on nationality, sex, and age, data of 170 people from the Dutch general population and data of 80 people from the British general population were selected to compare scores between DTF patients and the general population. Four British patients were excluded from this analysis due to missing data regarding their age at the time of questionnaire completion. Dutch patients had a score of 77 for global health and a summary score of 87.2, whereas scores for the matched Dutch population were 78.7 and 89.8 for global health and the summary score, respectively. British patients (*n* = 8) had a score of 59.4 for global health and a summary score of 68.2, whereas scores for the matched British population were 60.2 and 76.7 for global health and the summary score, respectively (Table 3) [16]. Dutch participating patients scored lower on all functioning scales compared to the general Dutch population, although only the physical functioning score (*p*=0.019) and the role functioning score (*p*=0.021) showed a statistically significant difference (Table 3). No statistically significant differences were found comparing EORTC QLQ-C30 scores between the British patients and the British general population.

## 4. Discussion

The purpose of this study was to identify the most important HRQoL issues for patients with sporadic DTF and rank them according to relevance. The most highly ranked HRQoL issues by patients with DTF were related to the unpredictable disease trajectory of DTF. Additionally, issues regarding the rarity, aggressiveness, and the benign classification of DTF received high scores. From the patient perspective, this benign classification was seen as misleading, as DTF can display aggressive growth. In terms of the healthcare system, the benign disease classification, not being cancer, can have both pros and cons as it can have consequences for insurances and covering of expenses, depending on the country of residence. As the aforementioned items are not included in the EORTC QLQ-C30 questionnaire, a tailored DTF HRQoL tool could capture these issues. Physical symptoms such as pain, fatigue, and loss of muscle strength also received high priority scores of 2.4, 2.3, and 2.3, respectively. Although these items are covered by the EORTC QLQ-C30 questionnaire, the results of this study highlight the importance of physical symptoms, caused by the tumour or as a side effect of treatment, and their impact on HRQoL. Patients identified several important issues that were not covered by other questionnaires. These could be considered in the development of a future DTF-specific HRQoL tool.

In a rare and heterogeneous disease, such as DTF, measuring the impact of the disease on patients can be challenging. This can be due to the variable disease presentation, course, and response to treatment and due to the knowledge gap of the natural history of the disease [20]. Moreover, the limited number of responses challenges research in this field. Our cohort may not be representative of the entire DTF population as the majority of patients in this cohort had an intra-abdominal tumour and many patients received one or multiple “active” forms of treatment.

In addition to physical, emotional, and psychological problems, patients with DTF might also experience social isolation due to lack of peers with the same condition [20]. This was reflected in the current study by a relatively high score for the issue “not knowing peers with the same disease.” Furthermore, lack of information was identified as a relevant topic as the following issues: “DTF is unknown among most doctors” and “lack of information received about DTF” received *M*-scores of 2.6 and 1.8, respectively.

HCPs may treat a limited number of patients with this rare disease; therefore, patients may receive an incorrect diagnosis or delay in diagnosis due to lack of experience in recognizing and treating the disease [20]. The comparison in relevance scores between patients and HCPs shows that HCPs scored significantly higher on a large number of issues, suggesting that they recognize and acknowledge problems faced by this patient group. The issue “reaching a definite diagnosis is time consuming” received an *M*-score of 2.3, showing that this is a relevant problem for this patient group. Whilst the future DTF-specific HRQoL tool will be available upon diagnosis, it is important for HCPs to consider that patients may have encountered difficulties reaching the correct diagnosis and so provision of clear information and support at this time is essential. Accessing specialists with knowledge of DTF can be challenging, as they may be located in regional specialist centres. This can result in patients receiving multiple treatment recommendations before seeing a specialist.

Financial consequences, due to insurance problems, the need to take time off work or increasing traveling costs can also affect HRQoL, although the issues regarding these subjects all received relatively low scores in the current study [21]. Social problems, such as the burden of having a rare disease on family and carers, as well as having this diagnosis at a young age, can also have a negative impact on HRQoL [20, 21].

This unique study identified important issues for DTF patients and compared the views of British and Dutch patients. Most issues were scored higher by British patients compared to Dutch patients (indicating a higher relevance for the specific issue). This phenomenon was also seen comparing EORTC QLQ-C30 scores, as British patients scored statistically significantly lower (indicating worse functioning) on four out of five function scales, and for the symptom scales insomnia, pain, and fatigue. Although both participating centres are tertiary centres visited by patients with more complex or advanced disease, the catchment area of the Royal Marsden Hospital (London, UK) is larger than that of the Erasmus MC (Rotterdam, NL) possibly creating selection bias during this study. Norm data obtained by the EORTC of the general Dutch and British population showed a comparable trend with higher scores on symptom scales and single items scales (indicating greater symptom burden) and lower scores on functioning scales (indicating worse functioning) comparing the data from the general Dutch and British population. Data from 2017 of The Organisation for Economic Co-operation and Development show similar results with lower scores (indicating a lower well-being) of British participants compared to Dutch participants on several measures of well-being (e.g., housing, income, education, and health and life satisfaction) [22]. This suggests that although our data might show differences between both countries of “impact of disease” on HRQoL, baseline scores in the normal population differ and that the experience of HRQoL issues depends on where you live [16, 22].

Comparisons between patients and a matched cohort of the general population based on nationality, sex, and age did not yield significant results, except for “physical functioning” and “role functioning” comparing the Dutch patients with the Dutch general population. Additionally, we compared the scores of HCPs and participating patients. An important finding of this study was the clear overlap of issues that were important to patients and HCPs. The HCPs rated various issues higher than patients particularly with regard to pain, stress about the diagnosis, and concerns about the future.

We acknowledge that this study has several limitations. The small sample size is explained by the rarity of DTF. A larger cohort is needed to test the psychometric aspects of a DTF-specific HRQoL tool in future studies. The response rate was lower than we had hoped for, but similar response rates have been published in studies describing more common diseases such as cancer [23]. In the current study, the relatively low response rate may have been due to the length of the questionnaire, the single-centre setup (one centre in each country), the timing of sending out the questionnaire (midsummer), and/or the overall reluctance to participate in a survey study. Furthermore, many patients also need to complete questionnaires as part of their regular healthcare; therefore, patients might be less willing to complete questionnaires for research purposes. Sending out a reminder to patients would have been a valid option to increase the response rate. Selection bias may have led to an overestimation of HRQoL problems in our cohort. As the primary aim was to identify the most relevant issues in this patient group, the effect of this overestimation is less relevant. A population-based cohort is required to determine the true prevalence of issues and perhaps a more representative result. Lastly, interpretation of the questions is influenced by the current health situation of each patient. We tried to eliminate such influencing factors by excluding patients with a diagnosis of cancer and FAP-associated DTF. However, patients HRQoL might also be influenced by disease stage, tumour location, and treatments and by other comorbidities and personal circumstances. This impact on HRQoL issues could be evaluated in a future population-based cohort study and stresses the need for validation of our findings in a large, international DTF cohort to evaluate the prevalence of HRQoL issues.

Today, solely one DTF-specific questionnaire, the Gounder/DTRF Desmoid Symptom/Impact Scale, is available and currently mainly used in the setting of clinical trials [24–26]. The findings of our study will be used for the development of a DTF-specific tool, according to the EORTC guidelines, which can be used, accompanied by the EORTC QLQ-C30 HRQoL instrument and will be useful for observational studies, clinical trials, and clinical care. Implementation of this tool and action on abnormal findings, concerns, or poor experiences of patients might improve satisfaction with healthcare, symptom management, and HRQoL [27]. Healthcare providers may benefit from being able to anticipate and identify problems earlier, thereby improving work efficiency and promoting patient-centred care through shared decision-making [28–30]. In order for a tailored HRQoL tool to work in clinical practice, this tool should add value to the clinical workflow without disrupting it [31]. Our results will be used in the development of an international, multicentre, population-based study in line with the EORTC guidelines for developing a questionnaire [14]. This study includes pretesting and content validation of a DTF-specific questionnaire. This questionnaire will assess the prevalence of HRQoL issues and will identify risk factors for the development of HRQoL issues patients experience. Patients will receive an invitation to participate in an online survey and one reminder for completing the questionnaire. When this tool has been developed and validated, it will reflect overall patient experience and its multidimensional contributing factors by including important nonsymptom, disease-specific areas regarding the unpredictable course of this rare disease. The tool could be used alongside the EORTC QLQ-C30, to gain more insight into HRQoL issues of the patient at diagnosis. Additionally, longitudinal studies could evaluate HRQoL issues of DTF patients during their disease trajectory, and the questionnaire can potentially be used in both clinical and research settings.

## 5. Conclusions

This study identified relevant issues for DTF patients to be considered in the future development of a DTF-specific HRQoL questionnaire. Issues regarding the unpredictable growth behaviour and rarity of DTF were the most highly ranked by patients and HCPs. Additionally, this study identified differences in priority scores between British and Dutch patients. Although this could be due to selection bias, field testing in a large, international cohort is needed to confirm any potential cultural findings.

## Figures and Tables

**Table 1:** Clinical characteristics of 29 participating patients.

		Total group (%)^a^		Dutch patients (*n* = 17)		British patients (*n* = 12)	
Sex	Male	10	(35%)	5	(29%)	5	(42%)
	Female	19	(65%)	12	(71%)	7	(59%)
Median age in years at the time of questionnaires (IQR)^b^		43	(36–55)	44	(36–55)	41	(32–56)
Median age in years at the time of diagnosis (IQR)^c^		38	(30–48)	38	(30–48)	37	(28–50)
Tumour localisation	Abdominal wall	2	(7%)	2	(12%)	0	(0%)
	Intra-abdominal	10	(35%)	8	(47%)	2	(17%)
	Extremity/girdles/chest wall	15	(52%)	6	(35%)	9	(75%)
	Head/neck/intrathoracic	1	(3%)	1	(6%)	0	(0%)
	Missing value	1	(3%)	0	(0%)	1	(6%)
Recurrent disease	Yes	6	(21%)	2	(12%)	4	(33%)
	No	21	(72%)	15	(88%	6	(50%)
	Missing value	2	(7%)	0	(0%)	2	(17%)
Received treatments (some patients gave multiple answers)	Wait and see	21		12		9	
	Surgery	14		8		6	
	Radiotherapy	4		1		3	
	Chemotherapy	5		1		4	
	Nonsteroidal anti-inflammatory drugs	8		1		7	
	Hormonal treatment	7		2		5	
	Pain management	9		0		9	
	Physiotherapy	7		3		4	
	Occupational therapy	2		1		1	
Currently receiving any active form of treatment	Yes	9	(31%)	0	(0%)	9	(75%)
	No	19	(66%)	17	(100%)	2	(17%)
	Missing value	1	(3%)	0	(0%)	1	(8%)
Comorbidity (some patients gave multiple answers)	No	11		6		5	
	Arthritis or long-term joint problem	3		2		1	
	Asthma or long-term chest problem	4		2		2	
	Diabetes	1		1		0	
	High blood pressure	1		0		1	
	Kidney or liver disease	1		1		0	
	Long-term back problem	6		3		3	
	Long-term mental health problem	2		2		0	
	Long-term neurological problem	1		1		0	
	Physical disability	2		1		1
	Others^d^	3		2		1	
	Missing value	2		2		0	

^a^Percentages may not add up to 100% due to rounding up of decimals. ^b^Answered by *n* = 21 participating patients. ^c^Answered by *n* = 29 participating patients. ^d^Including digestive problems, coeliac disease lactose intolerance, and iron deficiency.

**Table 2:** Top 10 most important issues according to the number of participating patients (*n*).

	*n*	Prevalence ratio (%)
Participating patients (total *n* = 16)^a^		
Worries about tumour growth	10	62.5
Fear of the tumour growth and/or tumour growing into adjacent tissues or organs	9	56.3
Feeling that there is something in your body that does not belong there	7	43.8
Stress around check-ups during the follow-up	6	37.5
Pain	6	37.5
Reaching a definite diagnosis is time consuming	5	31.3
Not being able to sleep because of pain		

Feeling frustrated about the “benign” diagnosis with malignant features	5	31.3
	5	31.3
Desmoid-type fibromatosis is unknown among most doctors	5	31.3
Healthcare providers (total *n* = 31)		
Worries about tumour growth	17	54.9
Experience of uncertainty during the course of disease	12	38.7
Pain	11	35.5
Lack of optimal treatment options and/or uncertainty about preferred treatment	10	32.3
Concerns about the future		
	10	32.3

^a^ *n* = 13 participating patients failed to provide a top 10. The cutoff value for inclusion in the DTF-specific HRQoL questionnaire is a prevalence ratio of >30%.

**Table 3:** Results of the EORTC QLQ-C30 questionnaire (version 3.0) of patients and the general population.

	Total mean (SD) patients		British participating patients, *n* = 12 mean (SD)		Dutch participating patients, *n* = 17 mean (SD)		*p* value	Dutch general population, *n* = 170 mean (SD)		British general population, *n* = 80^a^ mean (SD)	
Dyspnoea	10.3	(23.7)	8.0	(15.1)	11.8	(28.7)	0.845	8.2	(19.1)	18.7	(11.8)
Insomnia	31	(38.8)	55.6	(38.4)	13.7	(29.0)	**0.004** ^**∗**^	20.8	(25.1)	37.9	(39.6)
Appetite loss	14.9	(26.1)	22.2	(21.7)	9.8	(28.3)	0.073	2.9	(11.9)	16.2	(24.8)
Constipation^a^	16.7	(32.1)	21.2	(37.3)	13.7	(29.0)	0.781	4.7	(13.7)	14.6	(30.9)
Diarrhoea	17.2	(30.4)	27.8	(39.8)	9.8	(19.6)	0.325	7.3	(17.9)	14.2	(45.2)
Financial difficulties	11.5	(24.0)	22.2	(29.6)	3.9	(16.2)	0.059	5.7	(18.9)	23.3	(34.5)
Nausea/vomiting	7.5	(17.6)	13.9	(24.4)	2.9	(8.8)	0.180	4.7	(13.7)	14.6	(28.8)
Pain	33.9	(36.0)	58.3	(37.3)	16.7	(23.6)	**0.004** ^**∗**^	16.2	(21.9)	29.4	(40.3)
Fatigue	31	(32.9)	49.1	(29.0)	18.3	(29.9)	**0.004** ^**∗**^	22.5	(22.4)	33.9	(32.2)
Cognitive functioning	79.9	(30.3)	65.3	(32.1)	90.2	(25.0)	**0.030** ^**∗**^	91.7	(15.7)	76.7	(29.5)
Emotional functioning	73.6	(32.4)	59.0	(33.6)	83.8	(28.2)	**0.021** ^**∗**^	84.3	(18.9)	67.3	(36.7)
Social functioning	77.6	(29.6)	58.3	(33)	91.2	(17.8)	**0.001** ^**∗**^	94.2	(14.9)	75.4	(38.8)
Physical functioning	75.2	(27.9)	67.8	(32.5)	80.3	(23.9)	0.394	92.5	(13.1)	80.2	(33.3)
Role functioning	71.8	(32.8)	52.8	(40.1)	85.3	(17.6)	**0.027** ^**∗**^	91.8	(19.5)	76.5	(38.8)
Global health status	68.7	(27.7)	56.9	(29.5)	77.0	(23.9)	**0.043** ^**∗**^	78.7	(18.2)	60.2	(34.6)
Summary score	**78.1**		**63.5**		**87.2**			**89.8**		**76.7**	

^a^Data missing from 1 British patient; ^*∗*^statistically significant difference. Mean scores with standard deviation (SD) are displayed for all scales of the EORTC QLQ-C30. The *p* value represents the comparison of the scores of the British participating patients versus the scores of the Dutch participating patients. Two-sided *p* < 0.05 was considered statistically significant.

## Data Availability

There are no supporting data available for the current manuscript.

## Supplementary Materials

Supplementary Materials Supplemental Figure 1: differences in score of more than 1 point between Dutch and British patients. Supplemental Table 1: sociodemographic characteristics of 29 participating patients. Supplemental Table 2: mean *M*-scores per issue ranked according to their relevance. Supplemental Table 3: missing issues and quotes from patients. Supplemental Table 4: general remarks and quotes from patients.
