# Supplementary material for: Assessing the Desmoid-Type Fibromatosis Patients' Voice: Comparison of Health-Related Quality of Life Experiences from Patients of Two Countries
Source: Sarcoma. 2020 Jul 26;2020:2141939. doi: 10.1155/2020/2141939 (PMC7399762; doi:10.1155/2020/2141939)
Supplement: Supplementary Materials — Supplemental Figure 1: differences in score of more than 1 point between Dutch and British patients. Supplemental Table 1: sociodemographic characteristics of 29 participating patients. Supplemental Table 2: mean M-scores per issue ranked according to their relevance. Supplemental Table 3: missing issues and quotes from patients. Supplemental Table 4: general remarks and quotes from patients. [file 2141939.f1.docx]

**Supplemental Files**


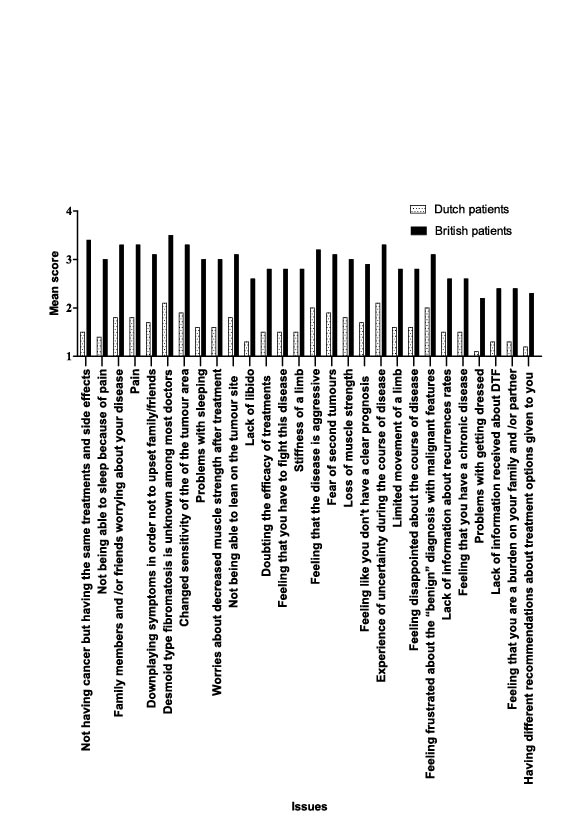
**Supplemental Figure 1.** Differences in score of more than 1 point between Dutch and British patients

| **Supplemental Table 1.** Sociodemographic characteristics of 29 participating patients | | | | | | | | |
| --- | --- | --- | --- | --- | --- | --- | --- | --- |
|  | | **Total group (%)^a^** | | | **Dutch patients**  **(n=17)** | | **British patients (n=12)** | |
| **Marital status** | Single  Dating /in a relationship  Married  Living common  Widowed  Separated  Divorced | 5  7  10  4  1  1  1 | (17%)  (24%)  (35%)  (14%)  (3%)  (3%)  (3%) | 1  5  5  3  1  1  1 | | (6%)  (29%)  (29%)  (18%)  (6%)  (6%)  (6%) | 4  2  5  1  0  0  0 | (33%)  (17%)  (42%)  (8%)  (0%)  (0%)  (0%) |
| **Current living situation** | Living with child(ren) and spouse /partner  Living with child(ren)  Living with spouse /partner  Living alone  Living with parent(s)  Missing value | 14  2  6  5  1  1 | (48%)  (7%)  (21%)  (17%)  (3%)  (3%) | 8  2  4  2  0  1 | | (47%)  (12%)  (24%)  (12%)  (0%)  (6%) | 6  0  2  3  1  0 | (50%)  (0%)  (17%)  (25%)  (8%)  (0%) |
| **Caring responsibilities for children (<18 years)** | Yes  No | 14  15 | (48%)  (52%) | 9  8 | | (53%)  (47%) | 5  7 | (42%)  (58%) |
| **Highest completed education** | High education  Intermediate education  Low education | 16  11  2 | (55%)  (38%)  (7%) | 10  5  2 | | (59%)  (29%)  (12%) | 6  6  0 | (50%)  (50%)  (0%) |
| **Current employment status** | Employed full-time (including self-employed)  Employed part-time (including self-employed)  Unemployed  Permanently unable to work  Retired | 14  6  2  6  1 | (48%)  (21%)  (7%)  (21%)  (3%) | 7  4  2  3  1 | | (41%)  (24%)  (12%)  (18%)  (6%) | 7  2  0  3  0 | (58%)  (17%)  (0%)  (25%)  (0%) |
| **Physical demanding job** | Not applicable  No physical demanding job  Physically light  Physically medium | 12  5  7  5 | (41%)  (17%)  (24%)  (17%) | 8  2  4  3 | | (47%)  (12%)  (24%)  (18%) | 4  3  3  2 | (33%)  (25%)  (25%)  (17%) |
| **Mentally challenging job** | Not applicable  Not at all challenging  Rarely challenging  Occasionally challenging  Frequently challenging  Constantly challenging | 9  1  1  8  7  3 | (31%)  (3%)  (3%)  (28%)  (24%)  (10%) | 6  1  0  4  4  2 | | (35%)  (6%)  (0%)  (24%)  (24%)  (12%) | 3  0  1  4  3  1 | (25%)  (0%)  (8%)  (33%)  (25%)  (8%) |
| *^a^ Percentages may not add up to 100% due to rounding up of decimals* | | | | | | | | |

| **Supplemental Table 2.** Mean (M) scores per issue ranked according to their relevance | | | | | | | | | | |
| --- | --- | --- | --- | --- | --- | --- | --- | --- | --- | --- |
| **Issues** | | **Total**  **Patients**  **(n=29)** | **Total**  **HCPs**  **(n=31)** | **p-value** | **Dutch patients**  **(n=17)** | **British patients**  **(n=12)** | **p-value** | **Dutch HCPs**  **(n=21)** | **British HCPs**  **(n=10)** | **p-value** |
| 1 | Worries about tumour growth | 3.0 | 3.4 | 0.186 | 2.6 | 3.5 | 0.020* | 3.2 | 3.8 | 0.172 |
| 2 | Feeling that there is something in your body that does not belong there | 2.9 | 2.8 | 0.889 | 2.5 | 3.3 | 0.066 | 2.7 | 3.1 | 0.287 |
| 3 | Fear of tumour growth/fear of the tumour growing into adjacent tissues or organs | 2.8 | 2.9 | 0.572 | 2.4 | 3.3 | 0.066 | 2.7 | 3.4 | 0.039***** |
| 4 | Desmoid type fibromatosis is unknown among most doctors | 2.6 | 2.8 | 0.453 | 2.1 | 3.5 | 0.001* | 2.7 | 3.2 | 0.209 |
| 5 | Stress around check-ups during the follow-up | 2.6 | 3.0 | 0.071 | 2.6 | 2.5 | 0.926 | 3.1 | 2.8 | 0.237 |
| 6 | Contemplation about the disease | 2.6 | 2.8 | 0.404 | 2.4 | 2.8 | 0.227 | 2.8 | 2.8 | 0.846 |
| 7 | Experience of uncertainty during the course of disease | 2.6 | 3.2 | 0.039* | 2.1 | 3.3 | 0.011* | 3.1 | 3.3 | 0.533 |
| 8 | Feeling that the disease is aggressive | 2.5 | 2.8 | 0.237 | 2.0 | 3.2 | 0.008* | 2.6 | 3.4 | 0.005***** |
| 9 | Change in life perspective (e.g. feeling thankful not having cancer) | 2.5 | 2.2 | 0.314 | 2.4 | 2.6 | 0.711 | 2.1 | 2.4 | 0.287 |
| 10 | Fear of recurrence after treatment | 2.5 | 3.0 | 0.063 | 2.1 | 3.1 | 0.014* | 3.0 | 3.0 | 1.000 |
| 11 | Family members and/or friends worrying about your disease | 2.5 | 2.4 | 0.776 | 1.8 | 3.3 | 0.000* | 2.0 | 3.0 | 0.010***** |
| 12 | Feeling frustrated about the “benign” diagnosis with malignant features | 2.4 | 2.9 | 0.203 | 2.0 | 3.1 | 0.018* | 2.7 | 3.2 | 0.205 |
| 13 | Changed sensitivity of the of the tumour area | 2.4 | 2.7 | 0.375 | 1.9 | 3.3 | 0.011* | 2.4 | 3.3 | 0.011***** |
| 14 | Pain | 2.4 | 3.2 | 0.016* | 1.8 | 3.3 | 0.002* | 2.9 | 3.9 | 0.007***** |
| 15 | Stress about the diagnosis | 2.4 | 3.2 | 0.007* | 2.5 | 2.3 | 0.677 | 3.2 | 3.3 | 0.530 |
| 16 | Fear of second tumours | 2.4 | 2.3 | 0.948 | 1.9 | 3.1 | 0.003* | 2.2 | 2.6 | 0.266 |
| 17 | Concerns about the future | 2.4 | 3.0 | 0.012* | 2.1 | 2.8 | 0.053 | 2.8 | 3.4 | 0.142 |
| 18 | Not being able to lean on the tumour site | 2.4 | 2.3 | 0.994 | 1.8 | 3.1 | 0.013* | 2.0 | 3.0 | 0.011***** |
| 19 | Fatigue | 2.3 | 2.4 | 0.687 | 2.0 | 2.8 | 0.122 | 2.1 | 3.0 | 0.022***** |
| 20 | Downplaying symptoms in order not to upset family/friends | 2.3 | 2.1 | 0.721 | 1.7 | 3.1 | 0.002* | 2.0 | 2.3 | 0.287 |
| 21 | Reaching a definite diagnosis is time consuming | 2.3 | 2.7 | 0.115 | 2.0 | 2.7 | 0.152 | 2.6 | 2.9 | 0.448 |
| 22 | Not having cancer but having the same treatments and side effects | 2.3 | 2.8 | 0.083 | 1.5 | 3.4 | 0.000* | 2.6 | 3.1 | 0.183 |
| 23 | Loss of muscle strength | 2.3 | 2.3 | 0.718 | 1.8 | 3.0 | 0.009* | 1.9 | 3.3 | 0.000***** |
| 24 | Reassurance because of the benign tumour | 2.2 | 2.5 | 0.214 | 2.4 | 2.0 | 0.318 | 2.5 | 2.6 | 0.929 |
| 25 | Feeling exhausted | 2.2 | 2.2 | 0.923 | 1.8 | 2.8 | 0.042* | 2.0 | 2.8 | 0.050 |
| 26 | Problems with playing sports | 2.2 | 2.9 | 0.019* | 2.0 | 2.5 | 0.264 | 2.7 | 3.3 | 0.055 |
| 27 | Feeling like you don’t have a clear prognosis | 2.2 | 2.9 | 0.015* | 1.7 | 2.9 | 0.003* | 2.7 | 3.2 | 0.266 |
| 28 | Fear of treatment | 2.2 | 2.5 | 0.235 | 2.1 | 2.4 | 0.444 | 2.2 | 3.2 | 0.013***** |
| 29 | Lack of optimal treatment options and/or uncertainty about preferred treatment | 2.2 | 2.8 | 0.039* | 1.8 | 2.7 | 0.026* | 2.5 | 3.3 | 0.055 |
| 30 | Life changing disease in a negative manner | 2.2 | 2.7 | 0.021* | 1.8 | 2.7 | 0.059 | 2.5 | 3.2 | 0.059 |
| 31 | Problems with sleeping | 2.2 | 2.3 | 0.528 | 1.6 | 3.0 | 0.003* | 2.0 | 2.9 | 0.006***** |
| 32 | Worries about decreased muscle strength after treatment | 2.2 | 2.3 | 0.502 | 1.6 | 3.0 | 0.004* | 2.0 | 2.8 | 0.019***** |
| 33 | Not knowing peers with the same disease | 2.1 | 2.6 | 0.068 | 1.9 | 2.5 | 0.329 | 2.3 | 3.3 | 0.013***** |
| 34 | Deterioration in physical fitness | 2.1 | 2.2 | 0.812 | 1.7 | 2.7 | 0.030* | 1.7 | 3.3 | 0.000***** |
| 35 | Feeling asymmetrical/misshaped | 2.1 | 2.3 | 0.284 | 1.8 | 2.5 | 0.059 | 2.0 | 3.1 | 0.001***** |
| 36 | Limited movement of a limb | 2.1 | 2.9 | 0.004* | 1.6 | 2.8 | 0.007* | 2.6 | 3.5 | 0.013***** |
| 37 | Continuous worries about the disease | 2.1 | 2.7 | 0.005* | 1.8 | 2.5 | 0.053 | 2.5 | 3.1 | 0.031***** |
| 38 | Trying to relieve the burden on family members | 2.1 | 2.2 | 0.423 | 1.8 | 2.4 | 0.227 | 2.1 | 2.6 | 0.169 |
| 39 | Feeling disappointed about the course of disease | 2.1 | 2.8 | 0.012* | 1.6 | 2.8 | 0.018* | 2.5 | 3.6 | 0.003***** |
| 40 | Doubting the efficacy of treatments | 2.0 | 2.8 | 0.011* | 1.5 | 2.8 | 0.008* | 2.5 | 3.4 | 0.019***** |
| 41 | Not being able to sleep because of pain | 2.0 | 2.7 | 0.021* | 1.4 | 3.0 | 0.001* | 2.2 | 3.7 | 0.000***** |
| 42 | Frustrated having to explain the meaning of this disease to others | 2.0 | 2.5 | 0.047* | 1.6 | 2.6 | 0.048* | 2.4 | 2.8 | 0.349 |
| 43 | Feeling that you have to fight this disease | 2.0 | 2.3 | 0.169 | 1.5 | 2.8 | 0.004* | 2.1 | 2.8 | 0.039***** |
| 44 | Stiffness of a limb | 2.0 | 2.5 | 0.056 | 1.5 | 2.8 | 0.004* | 2.3 | 3.1 | 0.048***** |
| 45 | Lack of information about recurrences rates | 2.0 | 2.5 | 0.003* | 1.5 | 2.6 | 0.013* | 2.2 | 3.2 | 0.019* |
| 46 | Covering up tumour(s) and/or scar(s) | 2.0 | 2.4 | 0.031* | 1.8 | 2.2 | 0.505 | 2.2 | 2.6 | 0.186 |
| 47 | Poor communication between GP’s and specialist, and also between professionals | 1.9 | 2.1 | 0.297 | 1.6 | 2.4 | 0.080 | 2.0 | 2.5 | 0.281 |
| 48 | Feeling that you have to explain your circumstances | 1.9 | 2.3 | 0.088 | 1.6 | 2.5 | 0.033* | 2.0 | 2.9 | 0.025* |
| 49 | Feeling that you have a chronic disease | 1.9 | 2.8 | 0.003* | 1.5 | 2.6 | 0.035* | 2.7 | 3.1 | 0.209 |
| 50 | Negative responses to appearance | 1.9 | 2.5 | 0.005* | 1.8 | 2.0 | 0.499 | 2.1 | 3.2 | 0.002* |
| 51 | Missing psychological support | 1.9 | 2.3 | 0.019* | 1.5 | 2.4 | 0.053 | 2.1 | 2.7 | 0.096 |
| 52 | Lack of libido | 1.8 | 2.0 | 0.208 | 1.3 | 2.6 | 0.005* | 1.8 | 2.5 | 0.025* |
| 53 | Having to take sick leave | 1.8 | 2.4 | 0.008* | 1.6 | 2.1 | 0.286 | 2.1 | 3.0 | 0.004* |
| 54 | Change of career ambitions after the diagnosis | 1.8 | 2.0 | 0.144 | 1.4 | 2.4 | 0.016* | 1.9 | 2.4 | 0.214 |
| 55 | Lack of continuity of doctors during follow-up | 1.8 | 2.2 | 0.058 | 1.4 | 2.4 | 0.089 | 1.9 | 2.8 | 0.035* |
| 56 | Lack of information received about DTF | 1.8 | 2.6 | 0.003* | 1.3 | 2.4 | 0.016* | 2.4 | 2.9 | 0.234 |
| 57 | Long distance to travel to the hospital | 1.8 | 2.4 | 0.002* | 1.5 | 2.2 | 0.208 | 2.2 | 3.0 | 0.063 |
| 58 | Short temper | 1.8 | 1.9 | 0.329 | 1.5 | 2.2 | 0.122 | 1.6 | 2.7 | 0.002* |
| 59 | Lack of confidence | 1.7 | 2.4 | 0.006* | 1.5 | 2.0 | 0.329 | 2.0 | 3.1 | 0.009* |
| 60 | Problems with concentration | 1.7 | 1.9 | 0.247 | 1.6 | 1.9 | 0.378 | 1.6 | 2.6 | 0.005* |
| 61 | Feeling that you are a burden on your family and/or partner | 1.7 | 2.4 | 0.007* | 1.3 | 2.4 | 0.025* | 2.1 | 2.9 | 0.019* |
| 62 | Negative body image | 1.7 | 2.7 | 0.000* | 1.6 | 1.9 | 0.404 | 2.3 | 3.7 | 0.000* |
| 63 | Forgetfulness | 1.7 | 1.5 | 0.602 | 1.5 | 2.0 | 0.147 | 1.2 | 2.2 | 0.004* |
| 64 | Worries about possible inheritance/passing condition to children | 1.7 | 1.9 | 0.079 | 1.4 | 2.1 | 0.161 | 1.6 | 2.5 | 0.019* |
| 65 | Difficulty explaining the disease to others | 1.7 | 2.6 | 0.001* | 1.3 | 2.3 | 0.082 | 2.5 | 2.8 | 0.504 |
| 66 | Problems with activities of daily living (e.g. washing/dressing) | 1.7 | 2.4 | 0.001* | 1.4 | 2.1 | 0.048* | 2.0 | 3.2 | 0.001* |
| 67 | Change in lifestyle | 1.7 | 2.3 | 0.034* | 1.4 | 2.3 | 0.022* | 2.1 | 2.7 | 0.022* |
| 68 | Not being able to enjoy hobbies | 1.6 | 2.5 | 0.001* | 1.3 | 2.2 | 0.007* | 2.1 | 3.2 | 0.147 |
| 69 | Problems with receiving treatment(s) in a cancer hospital | 1.6 | 2.2 | 0.014* | 1.6 | 1.7 | 1.000 | 2.0 | 2.7 | 0.150 |
| 70 | Not being able to work at all | 1.6 | 2.4 | 0.000* | 1.4 | 1.9 | 0.403 | 2.2 | 2.9 | 0.039* |
| 71 | Change of hair colour | 1.6 | 1.4 | 0.245 | 1.8 | 1.4 | 0.941 | 1.2 | 1.8 | 0.031* |
| 72 | Feeling that you are standing still in life | 1.6 | 2.4 | 0.003* | 1.4 | 2.0 | 0.329 | 2.1 | 2.9 | 0.065 |
| 73 | Feeling less feminine/feeling less masculine | 1.6 | 2.0 | 0.027* | 1.4 | 1.9 | 0.147 | 1.7 | 2.7 | 0.002* |
| 74 | Feeling depressed | 1.6 | 2.7 | 0.001* | 1.4 | 2.0 | 0.161 | 2.3 | 3.5 | 0.001* |
| 75 | Having different recommendations about treatment options given to you | 1.6 | 2.6 | 0.001* | 1.2 | 2.3 | 0.047* | 2.5 | 2.7 | 0.681 |
| 76 | Fear of dying | 1.6 | 2.1 | 0.030* | 1.6 | 1.5 | 0.863 | 1.7 | 2.9 | 0.002* |
| 77 | Lack of understanding from family members and/or friends | 1.6 | 2.3 | 0.002* | 1.4 | 1.9 | 0.140 | 2.0 | 3.0 | 0.007* |
| 78 | Being addicted to pain medication | 1.6 | 2.2 | 0.003* | 1.2 | 2.2 | 0.117 | 1.9 | 2.8 | 0.006* |
| 79 | Changing jobs | 1.5 | 2.3 | 0.001* | 1.4 | 1.8 | 0.285 | 2.0 | 3.0 | 0.017* |
| 80 | Feeling depressed | 1.5 | 2.4 | 0.000* | 1.4 | 1.8 | 0.134 | 2.0 | 3.2 | 0.001* |
| 81 | Problems with getting dressed | 1.5 | 1.8 | 0.048* | 1.1 | 2.2 | 0.003* | 1.6 | 2.4 | 0.007* |
| 82 | Breathless on minimal exertion | 1.5 | 1.7 | 0.286 | 1.4 | 1.7 | 0.505 | 1.3 | 2.5 | 0.000* |
| 83 | Being less independent | 1.5 | 2.2 | 0.001* | 1.1 | 2.1 | 0.037* | 1.9 | 3.0 | 0.004* |
| 84 | Fear of amputation of a limb | 1.5 | 2.1 | 0.005* | 1.4 | 1.6 | 0.817 | 1.9 | 2.6 | 0.077 |
| 85 | Weight loss | 1.5 | 1.5 | 0.277 | 1.5 | 1.5 | 0.890 | 1.3 | 2.0 | 0.017* |
| 86 | Problems walking | 1.5 | 2.2 | 0.005* | 1.4 | 1.7 | 0.353 | 1.8 | 3.0 | 0.002* |
| 87 | Feeling that you are wasting the time of cancer professionals | 1.5 | 1.5 | 0.284 | 1.1 | 2.1 | 0.088 | 1.3 | 2.0 | 0.039* |
| 88 | Negative influence of the disease on wishes to become a parent | 1.5 | 2.4 | 0.000* | 1.2 | 1.9 | 0.264 | 2.3 | 2.7 | 0.348 |
| 89 | Feelings of isolation | 1.5 | 2.0 | 0.007* | 1.1 | 2.0 | 0.042* | 1.6 | 2.9 | 0.000* |
| 90 | Altered nutritional intake | 1.5 | 1.4 | 0.615 | 1.2 | 1.8 | 0.100 | 1.3 | 1.7 | 0.082 |
| 91 | Lack of information about treatment side effects | 1.5 | 2.3 | 0.000* | 1.1 | 2.0 | 0.015* | 2.1 | 2.6 | 0.131 |
| 92 | Feeling disabled | 1.4 | 2.4 | 0.000* | 1.2 | 1.8 | 0.286 | 2.0 | 3.2 | 0.003* |
| 93 | Negative impact of the disease on family (life) | 1.4 | 2.5 | 0.000* | 1.2 | 1.8 | 0.187 | 2.2 | 3.3 | 0.002* |
| 94 | Being upset because of referral to a cancer hospital | 1.4 | 2.4 | 0.000* | 1.7 | 1.0 | 0.023* | 2.3 | 2.6 | 0.429 |
| 95 | Problems sitting down | 1.4 | 2.1 | 0.000* | 1.2 | 1.7 | 0.458 | 2.0 | 2.4 | 0.105 |
| 96 | Missing online support (forum/chat group) | 1.4 | 2.2 | 0.000* | 1.3 | 1.6 | 0.611 | 2.1 | 2.6 | 0.164 |
| 97 | Financial problems because you have had to change your job | 1.4 | 2.1 | 0.002* | 1.2 | 1.8 | 0.074 | 1.9 | 2.8 | 0.040* |
| 98 | Hair loss | 1.4 | 1.4 | 0.245 | 1.5 | 1.3 | 0.781 | 1.3 | 1.8 | 0.045* |
| 99 | Missing out on social occasions | 1.4 | 2.2 | 0.000* | 1.1 | 1.9 | 0.002* | 1.9 | 2.9 | 0.005* |
| 100 | Financial problems due to travel expenses | 1.4 | 2.1 | 0.001* | 1.2 | 1.7 | 0.074 | 1.7 | 3.0 | 0.002* |
| 101 | Marital/relational problems | 1.4 | 2.1 | 0.001* | 1.2 | 1.6 | 0.187 | 1.8 | 2.9 | 0.003* |
| 102 | Financial problems due to the disease or treatment(s) | 1.4 | 2.3 | 0.000* | 1.1 | 1.8 | 0.155 | 2.1 | 2.9 | 0.035* |
| 103 | Worries about tumour growth during pregnancy | 1.4 | 2.5 | 0.000* | 1.4 | 1.3 | 0.505 | 2.4 | 2.8 | 0.390 |
| 104 | Nausea | 1.4 | 1.5 | 0.296 | 1.1 | 1.7 | 0.111 | 1.2 | 2.2 | 0.001* |
| 105 | Feeling lonely | 1.4 | 2.0 | 0.008* | 1.1 | 1.8 | 0.012* | 1.6 | 2.9 | 0.001* |
| 106 | Change of dominant side during writing | 1.4 | 1.7 | 0.005* | 1.0 | 1.9 | 0.111 | 1.5 | 2.1 | 0.049* |
| 107 | Change in appetite | 1.3 | 1.8 | 0.019* | 1.1 | 1.6 | 0.042* | 1.5 | 2.4 | 0.002* |
| 108 | Oedema (abnormal accumulation of fluid) | 1.3 | 1.8 | 0.002* | 1.3 | 1.4 | 0.890 | 1.7 | 2.0 | 0.231 |
| 109 | Problems with fertility | 1.3 | 2.0 | 0.001* | 1.1 | 1.6 | 0.342 | 1.8 | 2.8 | 0.016* |
| 110 | Parental role affected | 1.3 | 2.2 | 0.000* | 1.3 | 1.3 | 0.522 | 1.9 | 2.8 | 0.048* |
| 111 | Vomiting | 1.3 | 1.5 | 0.106 | 1.1 | 1.6 | 0.141 | 1.2 | 1.9 | 0.008* |
| 112 | Eczema | 1.3 | 1.3 | 0.643 | 1.2 | 1.3 | 0.963 | 1.2 | 1.6 | 0.449 |
| 113 | Less attention from family and friends because of the “benign” disease | 1.2 | 2.1 | 0.000* | 1.1 | 1.5 | 0.370 | 1.8 | 2.7 | 0.013* |
| 114 | Lack of trust in your treating physician | 1.2 | 2.0 | 0.000* | 1.2 | 1.3 | 0.711 | 1.9 | 2.3 | 0.307 |
| 115 | Feeling guilty towards siblings because you receive more attention because of your disease | 1.2 | 1.6 | 0.004* | 1.1 | 1.4 | 0.611 | 1.4 | 1.9 | 0.114 |
| 116 | Shipped from hospital to hospital to hospital | 1.2 | 2.2 | 0.000* | 1.3 | 1.1 | 0.711 | 2.3 | 2.1 | 0.769 |
| 117 | Missing school | 1.2 | 2.1 | 0.000* | 1.3 | 1.1 | 0.897 | 1.8 | 2.7 | 0.014 |
| 118 | Feeling ashamed to use support tools (e.g. wheelchair, scooter, electric bike etc.) | 1.2 | 1.9 | 0.000* | 1.1 | 1.4 | 0.537 | 1.7 | 2.4 | 0.074 |
| 119 | Lack of support from family/friends | 1.2 | 1.9 | 0.000* | 1.2 | 1.2 | 0.941 | 1.7 | 2.3 | 0.048* |
| 120 | Suicidal thoughts | 1.2 | 1.6 | 0.009* | 1.2 | 1.2 | 0.643 | 1.2 | 2.6 | 0.000* |
| 121 | Problems with driving a car | 1.2 | 2.0 | 0.000* | 1.1 | 1.3 | 0.537 | 1.7 | 2.6 | 0.003* |
| 122 | Loss of friendships | 1.1 | 1.9 | 0.000* | 1.1 | 1.2 | 0.890 | 1.6 | 2.6 | 0.010* |
| 123 | Hives | 1.1 | 1.3 | 0.211 | 1.1 | 1.2 | 0.890 | 1.1 | 1.6 | 0.173 |
| 124 | Change of living accommodation | 1.1 | 1.7 | 0.001* | 1.0 | 1.2 | 0.414 | 1.3 | 2.4 | 0.001* |
| **Total mean** | | **1.8** | **2.3** |  | **1.5** | **2.2** |  | **2.0** | **2.8** |  |
| ** statistically significant difference; HCP, health care provider*  *Grey marked areas are issues with an M-score of >1.5 given by either patients or HCPs, the cut of value for inclusion in the DTF-specific HRQoL-tool. The p-value represents the comparison of the scores of the participating patients and HCPs, the comparison of the scores of the Dutch and British participating patients, and the comparison of the scores of Dutch and British HCPs.* | | | | | | | | | | |
|  | |  |  |  |  |  |  |  |  |  |

| **Supplemental Table 3.** Missing issues and quotes from patients | | |
| --- | --- | --- |
|  | **Missing issues** | **Related quotes** |
| **Financial Issues** | Questions about health care insurance and coverage for medical aids | - “It is not a ‘cancer’ and although benign, insurance companies won’t cover as it falls under sarcoma which they assume is cancer. Yet, we want to try new medicines and treatments but it is not cancer. I feel we don't get the same medical urgency. Sometimes you feel you wish it was cancer to get the help we need. We should be entitled to be discriminated against, either way, is how many of us are left feeling.” - “Feeling that you have to fight this disease, and the injustice surrounding it: e.g. life insurance” - “My health care insurance caused problems when I wanted a bigger breast prosthetic in my unaffected breast to make my breasts symmetrical” - “Restricted access to the National Health Service when professionals did not know whether to treat as a cancer or not. For example; I was entitled to free patient transport which is available to those with cancer (to my particular hospital) when I was trying to sort out transport for 25 straight days.” |
| **Information** | Adequate online information | - “Lack of trusted information about DTF online, or any research” - “I’m not a Facebook fan and I don’t use it for anything else, but I can’t tell you how much I have learned from reading about the experiences of others. And it is truly heart breaking when you see a newcomer to the site having plucked up the courage to post and say ‘help’ because they have a diagnosis and literally no information, support or route to other knowledge” |
|  | Treatment options outside the region or country | - “The only additional worry I have is certain treatment options are only available depending on where in the country you live. For example, one patient was offered cryotherapy in Manchester, UK, but that is not offered in London, UK.” |
|  | Interaction with other pre-existing conditions. For example: fatigue from pain in one condition seemed to set off the pain in the other |  |
| **Hobbies/leisure** | Problems with cycling |  |
|  | Lack of advice regarding eating or playing sports |  |
| **Health care facilities** | Pain management and referral to a pain specialist | - “Certainly my greatest issues are with pain management and effect this has on sleep and concerns for the future and what changes could happen at any time, positive and negative” |
| **Symptoms** | Intestinal problems e.g. diarrhoea or constipation, needing a stoma |  |
|  | Specific pain during certain movements (e.g. turning) |  |
|  | Trouble tying shoelaces |  |
|  | Extra tumour pain during menstruation |  |
|  | Constant worries | - “Constant trepidation about what it is going to do next, whether further growth” - “Feeling like a hypochondriac” - “The constant worry about ‘what is it doing now’ is always to the forefront, to the point that I started to keep a daily note |
|  |  | - during certain periods of what felt like the same feelings when it was very actively growing” |

| **Supplemental Table 4.** General remarks and quotes from patients | |
| --- | --- |
| **Remarks** | **Quotes** |
| Difficult to interpret some of the questions since many issues change over time |  |
| Due to multiple recurrences; ‘fear for a new tumour rather than recurrent tumours’ |  |
| Psychological support at the beginning of the disease | - “I would have loved at the beginning is psychological support. Being a very active and confident person, adjusting to initial limitations was very hard and I really struggled both physically and mentally” |
| Some issues are caused by the operation rather than the tumour itself which is difficult to distinguish from each other |  |
| Having a benign tumour which is aggressive, but no cancer | - “The biggest problem is having an aggressive recurrent tumour treated by oncologist with cancer treatment being less than cancer” - “It’s benign so it’s not going to kill you; the only thing it could do to kill you would be to suffocate you if it overwhelms your organs” |
| A good band with your treating physician | - “I learned thinking positive from my treating physician” |
| Fear of tumour growth during and after pregnancy | - “Especially post pregnancy with fear of further desmoid developing from scar from the caesarean section” |
